# The Prowess of Andrographolide as a Natural Weapon in the War against Cancer

**DOI:** 10.3390/cancers12082159

**Published:** 2020-08-04

**Authors:** Ammad Ahmad Farooqi, Rukset Attar, Uteuliyev Yerzhan Sabitaliyevich, Nada Alaaeddine, Damião Pergentino de Sousa, Baojun Xu, William C. Cho

**Affiliations:** 1 Department of Molecular Oncology, Institute of Biomedical and Genetic Engineering (IBGE), Islamabad 44000, Pakistan; farooqiammadahmad@gmail.com; 2 Department of Obstetrics and Gynecology, Yeditepe University, İstanbul 34755, Turkey; ruksetattar@hotmail.com; 3 Kazakhstan Medical University KSPH, Almaty 050004, Kazakhstan; yerzhan1810@gmail.com; 4 Neuroscience Research Center, Faculty of Medical Sciences, Lebanese University, Beirut 00961, Lebanon; Nada.aladdin@gmail.com; 5 Department of Pharmaceutical Sciences, Universidade Federal da Paraíba, João Pessoa 58051-970, Brazil; damiao_desousa@yahoo.com.br; 6 Food Science and Technology Program, BNU-HKBU United International College, Zhuhai 519087, China; 7 Department of Clinical Oncology, Queen Elizabeth Hospital, Hong Kong, China

**Keywords:** andrographolide, oncogenic signaling pathways, TRAIL, anti-cancer drug

## Abstract

There has been a paradigm shift in our understanding about the multifaceted nature of cancer, and a wealth of information has revealed that single-target drugs are not good enough to provide satisfactory clinical outcomes and therapeutic effects for complex diseases which involve multiple factors. Therefore, there has been a reignition to search for natural products having premium pharmacological activities aim to efficiently target multiple deregulated cellular signaling pathways. Andrographolide, a diterpene lactone from *Andrographis paniculata* was brought into to the limelight because of its ability to inhibit cancer cell proliferation and induce apoptosis. Here we reviewed andrographolide on cellular pathways regulation including Wnt/β-catenin, mTOR, VEGF-mediated intracellular signaling, as well as TRAIL-mediated apoptosis to inhibit cancer development.

## 1. Introduction

Recent developments in functional genomics, proteomics and next-generation sequencing have revolutionized the fields of molecular and clinical oncology [1,2,3,4]. Scientific breakthroughs have helped researchers in dissecting the intertwined network of oncogenic signal transduction cascades [5,6,7,8,9]. The deregulation of spatio-temporally controlled cell signaling pathways plays fundamental role in enhancing the ability of cancer cells to survive under “drug pressure”, the induction of epithelial-to-mesenchymal transition (EMT), metastatic spread, invasion of distant organs, and loss of apoptosis [10,11]. 

Secondary metabolites from plants are a rich source of bioactive compounds and have clinical use against various diseases [12,13], e.g., the anticancer drugs podophyllotoxin, vinca alkaloids (vinblastine and vincristine), and camptothecin. Among these compounds, the terpenes constitute the largest class of natural products and are widely used in food and pharmaceutical industry [14]. A broad range of biological and pharmacological activities have been reported for compounds of this chemical class, such as anti-inflammatory [15] and antitumor [16,17] actions. Therefore, terpenes provide exciting avenues for the discovery of new drug candidates.

In fact, many members of terpene family have entered various phases of clinical trials and some of them have been incorporated into backbone of modern medicine, e.g., paclitaxel. This compound is a diterpene isolated from the bark of *Taxus brevifolia* Nutt. (Taxaceae) and produced on an industrial scale for clinical application. 

**Figure 1:**
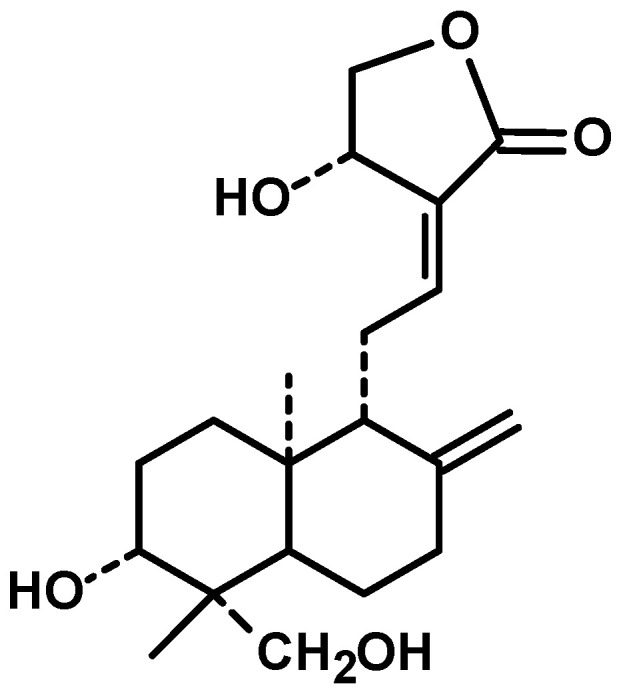
Chemical structure of andrographolide.

Diterpenes are constituents of four isoprenic units, they can be found in several medicinal plants and have various pharmacological properties, including antitumor [18]. *Andrographis paniculata* (Burm. f.) Nees, an annual herb from family Acanthaceae and cultivated in southern Asia, is a source of diterpenes [19]. Andrographolide (Figure 1) is a bicyclic diterpenoid lactone and main bioactive chemical constituent of this plant with remarkable antitumor activity. Other diterpenes structurally analogous to andrographolide are also present in the extract of *Andrographis paniculata*, such as neoandrographolide, homoandrographolide, and isoandrographolide [20]. 

Because of the noteworthy pharmacological features of andrographolide and encouraging evidence obtained from preclinical studies, andrographolide has also been tested for efficacy individually and combinatorially with capecitabine for the treatment of colorectal cancer (NCT01993472). The entry of andrographolide in clinical trial straightforwardly advocated its good potential.

Here we reviewed the important research findings of andrographolide. Spotlights on the abilities of andrographolide to modulate JAK-STAT, Wnt/β-Catenin, mTOR, and VEGF/VEGFR-driven signaling pathways are made. Besides, we also summarized about the studies on the restoration of the TRAIL-driven pathway by andrographolide in various cancers. Lastly, we analyzed the developments of non-coding RNAs and the knowledge gaps which have to be bridged for a better translation of andrographolide potential as an effective anticancer drug. 

## 2. Regulation of JAK-STAT Signaling

**Figure 2:**
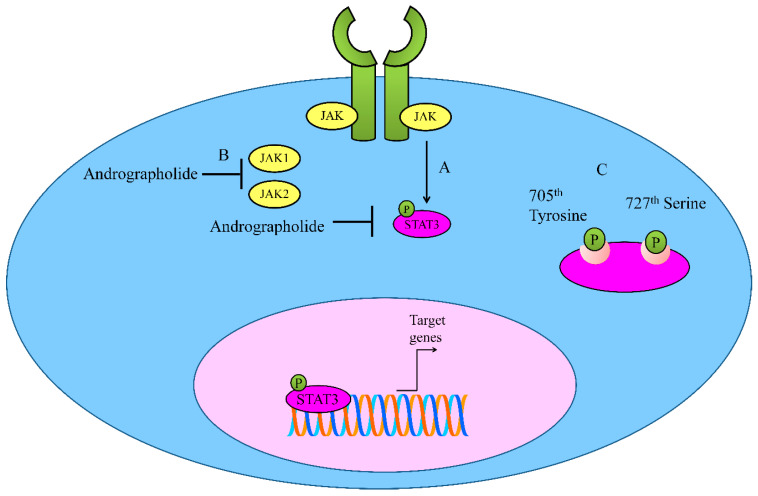
(**A**,**B**) Regulation of JAK-STAT signaling by Andrographolide. Andrographolide effectively inhibited JAK1, JAK2 and STAT3. (**C**) Andrographolide inhibited phosphorylation of STAT3 on 705th Tyrosine and 727th Serine.

The tightly orchestrated and spatio-temporally controlled JAK/STAT (Janus Kinase/Signal Transducer and Activator of Transcription) has been shown to play instrumental role in different cellular processes (Figure 2). The deregulation of the JAK-STAT pathway was noted to be involved in cancer development. STAT proteins are phosphorylated and consequently move into the nucleus to transcriptionally upregulate target genes [21,22,23]. SOCS (suppressor of cytokine signaling) and PIAS (protein inhibitors of activated STATs) are negative regulators of STAT-driven signaling [24]. However, the ability of andrographolide to regulate PIAS and SOCS in different cancers is still unclear. The regulation of STAT proteins by andrographolide has been investigated in different diseases. Here, we review the ability of andrographolide to modulate STAT proteins in cancers. 

Andrographolide inhibited interleukin-6 (IL-6) at mRNA and protein levels in a dose-dependent manner [25]. Andrographolide repressed IL-6 autocrine loop- and paracrine loop-driven signaling mainly through interfering with phosphorylation of STAT3 and ERK (extracellular signal regulated kinase). Mechanically, andrographolide induced apoptosis in castration-resistant and androgen-stimulated prostate cancer cells. Additionally, the intraperitoneal administration of andrographolide significantly reduced tumor growth in mice xenografted with DU145 cells [25].

Andrographolide inhibited constitutive phosphorylation of STAT3 on 705th Tyrosine and 727th Serine in a dose-and time-dependent manner [26]. It is reported that andrographolide efficiently inhibited IL-6-triggered STAT3 phosphorylation and consequent accumulation in the nucleus in HCT116 cells. Molecularly, andrographolide inhibited tyrosine phosphorylation of JAK1 and JAK2 [26]. 

Andrographolide was also found to be effective against pancreatic cancer cells [27]. IL-6 induced p-JAK2 and p-STAT3 in Panc-1 and AsPC-1 cells and treatment with andrographolide drastically suppressed IL-6-mediated p-JAK2 and p-STAT3. Andrographolide effectively reduced tumor volume in mice xenografted with AsPC-1 cells. It is interesting to see that andrographolide worked synergistically with gemcitabine to induce tumor regression in xenografted mice [27].

The development of STAT inhibitors is gradually gaining attention as an effective strategy to shut down target gene network controlled by STAT proteins. In accordance with this concept, OPB-31121 and OPB51602 had made entry into clinical trials and completed Phase I/II clinical trials for various cancers including, non-Hodgkin’s lymphoma, hepatocellular carcinoma (HCC), multiple myeloma and hematologic malignancies. Despite the fact that initial studies for OPB-31121 demonstrated efficacy against STAT3, but clinical trials in HCC showed minimal tumor inhibitory activity, poor pharmacokinetic features and toxicities associated with peripheral nervous system. Clinical trials for these inhibitors were terminated because of poor pharmacokinetic features and intolerability. Pyrimethamine, originally identified as an anti-malarial agent, is currently in phase I/II clinical trials for the treatment of small lymphocytic leukemia and chronic lymphocytic leukemia (https://clinicaltrials.gov/ct2/show/NCT01066663, accessed on 6 January 2020). Another stumbling block that needs to be overcome in context of STAT3 inhibitors is the relatively higher cytotoxic IC_50_ value, which limits further developments. In view of the existing challenges related to STAT inhibitors, it will be exciting to combine OPB-31121 or OPB-51602 with andrographolide in preclinical studies. 

## 3. Wnt/*β*-Catenin Pathway

Since the initial discovery of the oncogenic functionality of WNT1 in mouse mammary glands, scientists have gained a better understanding of multifaceted roles of WNT signaling cascade in different cancers [28,29]. WNTs and their downstream effectors modulate various processes which are essential for cancer progression, including tumor growth and metastatic spread. Canonical Wnt signaling revolves around the stabilization and nuclear accumulation of the transcriptional co-activator *β*-catenin [30].

Medicinal chemists have tested the pharmacological activities of andrographolide analogues. In accordance with this approach, 19-O-triphenylmethyl andrographolide (RS-PP-050), exerted inhibitory effects on TCF/LEF (T-cell factor and lymphocyte enhancing factor) activities in *β*-catenin-overexpressing cancer cells [20]. RS-PP-050 blocked the nuclear accumulation of phosphorylated *β*-catenin. Surprisingly, RS-PP-050 inhibited the activation of *β*-catenin independently of GSK-3*β*-directed mechanism [31].

Another analogue, 3A.1 (19-tert-butyldiphenylsilyl-8, 17-epoxy andrographolide), significantly inhibited TCF/LEF promoter activity [32]. Expression levels of *β*-catenin and Wnt-stimulated target genes were markedly reduced. Furthermore, analogue 3A.1 substantially enhanced function of GSK-3*β* which promoted *β*-catenin degradation. The analogue 3A.1 mediated effect was impaired after treatment with GSK-3*β* inhibitors or upon the over-expression of mutant *β*-catenin in colon cancer cells [32].

SOX9 was frequently overexpressed in poorly differentiated chondrosarcomas and SOX9 knockdown suppressed growth of chondrosarcoma cells [33]. Andrographolide dose dependently down-regulated SOX9 expression in chondrosarcoma cells. More importantly, andrographolide inhibited the mRNA expression of TCF-1 and simultaneously enhanced degradation of TCF-1 protein [33]. 

These findings collectively suggested that andrographolide interfered with activation and nuclear accumulation of *β*-catenin. More importantly, combinatorial treatment with *β*-catenin inhibitors or Wnt signaling inhibitors with andrographolide will be helpful in analysis of true potential of andrographolide as *β*-catenin inhibitor. 

CWP232291 is a peptidomimetic drug that targets *β*-catenin for degradation. It is currently being tested in combination with dexamethasone and lenalidomide in MM patients (NCT02426723), as monotherapeutic agent for patients with refractory or relapsed myeloid malignancies (NCT01398462) and combinatorially with cytarabine in relapsed and/or refractory acute myeloid leukemia patients (NCT03055286). It will be interesting to study tumor growth regression in xenografted mice inoculated with *β*-catenin-overexpressing cancer cells. Accordingly, peptidomimetics can be combined with andrographolide for analysis of synergistic effects.

## 4. Regulation of mTOR Pathway

mTOR (mammalian target of rapamycin), a serine/threonine kinase is a master regulator of metabolism and growth of the cells [34]. mTOR is a part of two structurally and functionally unique multi-component assemblies, mTOR complex 1 (mTORC1) and mTORC2 [35]. mTORC1 directly activated S6K (ribosomal protein S6 kinase) and inhibited 4EBP (eIF4E-binding protein) to increase translation. mTORC2 promoted metabolism mainly through AKT activation. 

Andrographolide significantly enhanced the levels of p-AMPK (AMP-activated Protein Kinase) and p-LKB1/STK11 (liver kinase B1 or serine/threonine kinase-11) [36]. Andrographolide also reduced the levels of p-P70S6K and p-S6 in nasopharyngeal carcinoma cells [36].

There is direct evidence showed the ability of andrographolide to induce autophagic cell death. Detailed mechanistic insights revealed that andrographolide significantly inhibited activation of AKT and mTOR in MG-63 and U-2OS osteosarcoma cells [37]. Interestingly, it was reported that andrographolide reduced phosphorylated-mTOR and AKT by generation of reactive oxygen species (ROS) [38]. A reduction in ROS levels interfered with ability of andrographolide to inactivate mTOR and AKT [38]. 

Andrographolide decreased the levels of phosphorylated-mTOR (Ser2481), whereas total mTOR levels remained unchanged [39]. Levels of RAPTOR and RICTOR, members of mTORC1 and mTORC2, respectively, were also found to be downregulated by andrographolide [39]. 

Andrographolide may prevent tumorigenesis in mice by inhibition of inflammation [40]. Andrographolide-induced mitophagy was found to be significantly involved in inactivation of NLRP3 inflammasomes and amelioration of animal models for colitis and colitis-associated cancer. More importantly, andrographolide inhibited the PIK3CA-AKT1-mTOR- P70S6K pathway [40]. 

Treatment of hypoxic MDA-MB-231 cells with andrographolide resulted in reduced HIF-1α expression along with a decrease in phosphorylated levels of AKT, mTOR, and its downstream effector P70S6K [41]. However, phosphorylation of 4EBP1 was markedly enhanced, which controlled the initiation of protein translation under hypoxic conditions [42]. 

**Figure 3:**
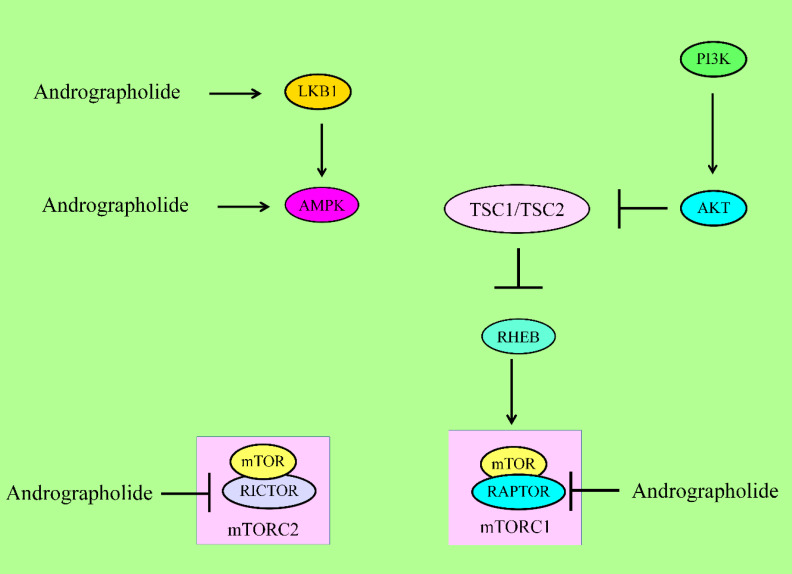
For activation of mTORC1, PIP3 recruited PDK1 to activate AKT. AKT inhibited the TSC complex, which acted as GTPase-activating protein (GAP) on the GTPase RHEB. GTP-bound RHEB structurally associated and activated mTORC1. Energy stress activated LKB1 and AMPK to suppress mTORC1 via inhibition of RAPTOR and activation of the TSC complex1. Andrographolide inhibited RAPTOR (mTORC1) and RICTOR (mTORC2). mTOR has different domains. RAPTOR and RICTOR bind to HEAT repeats of mTOR. Phosphoinositide-dependent kinase 1 (PDK1), liver kinase B1 (LKB1), AMP-activated kinase (AMPK), regulatory-associated protein of mTOR (RAPTOR).

On the other hand, andrographolide is also involved in suppression of autophagy mainly through activation of AKT/mTOR signaling (Figure 3). PTEN (Phosphatase and tensin homolog) is a negative regulator of AKT and dephosphorylates AKT [41]. Andrographolide induced the activation of AKT mainly through downregulation of PTEN [41]. 

Overall, these researches have provided clues that andrographolide dualistically regulated mTOR pathway in different cancers. 

## 5. Regulation of NLRP3 Inflammasome by Andrographolide

The activation of NLRP3 inflammasome occurred through deubiquitination of NLRP3, whereas inhibition of deubiquitination inhibited activation of NLRP3 [43,44]. Additionally, ASC (Apoptosis-associated Speck-like Protein containing a CARD) was noted to be ubiquitinated linearly for NLRP3 inflammasome assembly. Structural studies showed that NLRP3 NBD oligomerized the NLRP3 PYD (PYRIN domain), which served as molecular scaffolds for nucleation of ASC proteins. ASC and NLRP3 interacted structurally through PYRIN domains [43,44]. Consequently, ASC was converted into a prion-like structure and generated longer ASC filaments which were critical for the activation of inflammasomes. Pro-caspase-1 interacted with ASC through CARD (caspase recruitment domain) and formed its own prion-like filaments which branched off from the ASC filaments. Pro-caspase-1 was converted into molecularly functional caspase-1 [43,44].

Andrographolide inhibited activation of CASP1 [40]. Andrographolide inhibited secretion of interleukin-1B from lipopolysaccharide treated human monocytic THP-1 cells and murine bone marrow-derived macrophages (BMDM) in a dose-dependent manner. Likewise, the activity of CASP1 was significantly inhibited by andrographolide. Moreover, immunofluorescence and immunoprecipitation analysis provided evidence that andrographolide interfered with NLRP3 inflammasome formation [40].

## 6. Regulation of VEGF/VEGFR Signaling

Intracellular transduction of signals from the interaction of the family of vascular endothelial growth factors (VEGFs) and their receptors (VEGFRs) are central regulators of angiogenesis. 

Molecular-docking module and biochemical analyses have shown that andrographolide acts as a highly efficient docking molecule that binds effectively to ATP-binding pocket of VEGFR2 and interferes with its kinase activity [45].

Furthermore, 15-Benzylidene substituted derivatives (ADN-9) of andrographolide effectively induced regression of growth and metastasis of both orthotopically and subcutaneously xenografted tumors [46]. Additionally, ADN-9 exhibited stronger repressive effects on migratory capacity and VEGF-induced capillary-like tubular structure forming abilities of HUVECs mainly through interfering with VEGF/VEGFR2/AKT signaling pathway. Levels of phosphorylated-VEGFR2 were found to be notably reduced. Likewise, active AKT levels were reduced in VEGF-treated liver cancer cells [46].

**Figure 4:**
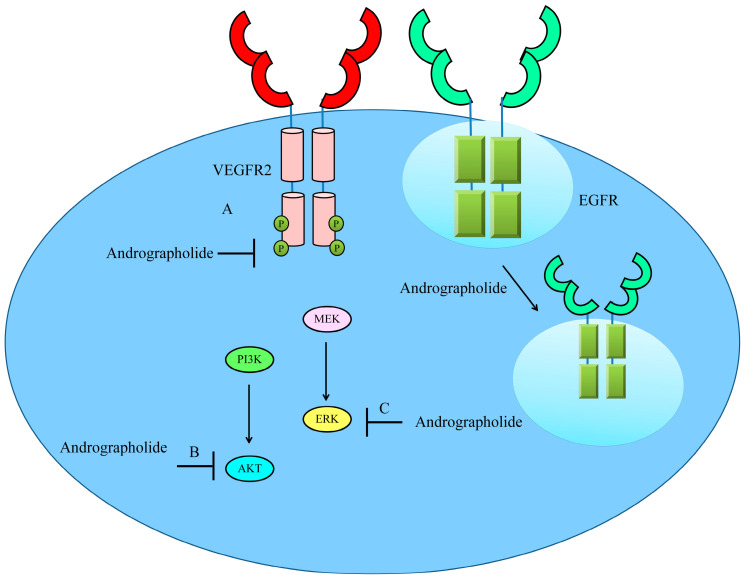
Schematic diagram of regulation of VEGF/VEGFR-driven pathway by andrographolide. Andrographolide effectively inhibited phosphorylation of VEGFR, AKT and ERK. Andrographolide also promoted internalization of EGFR to inhibit cell surface expression of EGFR and reduce EGF/EGFR-driven downstream signaling.

Andrographolide inhibited VEGFA-induced phosphorylation of VEGFR2 and different kinases present within signaling module particularly mitogen-activated protein kinases (MAPKs) [47]. Importantly, andrographolide interfered with the binding of VEGFA to VEGFR2. Andrographolide prevented VEGFA-directed activation and phosphorylation of VEGFR2 and MAPKs [47] (Figure 4).

Semi-synthetic andrographolide analogue A5 has also been tested previously against Hep3B cancer cells [48]. A5 was noted to be highly effective against ERK1/2 (extracellular signal regulated kinase-1/2) and p38 MAPK. In addition, A5 significantly blocked VEGF-triggered activation of VEGFR2 [48]. 

Semaxinib (SU5416) has been tested for efficacy in patients with metastatic colorectal carcinoma but SU5416-containing regimens did not show survival benefits, which consequentially resulted in the cessation of further developments of this compound [49]. Likewise, Vatalanib (PTK787/ZK 222584) also did not show remarkable clinical efficacy [50]. Therefore, andrographolide might be a promising candidate for the inhibition of a wide range of VEGFRs in different cancers.

## 7. Regulation of TRAIL Pathway

Basic and clinical scientists have paid much attention to the identification of molecules having maximum anticancer activity and minimum off-target effects. TRAIL (tumor necrosis factor-related apoptosis-inducing ligand) has surfaced as one of the highly efficient molecules reportedly involved in targeted killing of cancerous cells. TRAIL transduced the intracellular signals through death receptors (DR4 and DR5). Mechanistically, it has been shown that a decrease in cell surface expression of death receptors severely abrogated TRAIL mediated apoptosis [51].

These groundbreaking discoveries attracted pharmaceutical industries to design and develop different agents. Genentech developed dulanermin, a recombinant soluble human Apo2 ligand/TRAIL. Unfortunately, dulanermin failed to show significant overall therapeutic effects as a monotherapeutic agent [52]. Interdisciplinary research efforts were made to introduce more drugs with better efficacy. In accordance with this approach, agonistic antibodies against DR4 (mapatumumab) and DR5 (drozitumab, lexatumumab, conatumumab, LBY-135, and tigatuzumab) were tested for efficacy in clinical trials. 

Andrographolide significantly enhanced TRAIL-induced cleavage of FLIP-L and reduction of XIAP levels in HepG2 cells. Moreover, ectopic expression of either XIAP or FLIP-L severely impaired andrographolide-mediated TRAIL sensitizing effects [53]. Andrographolide considerably stimulated the expression of DR4. Excitingly, p53 was found to be essential for the DR4 upregulation. Results were further confirmed by analysis of expression levels of DR4 in andrographolide-treated p53 wild-type and mutant cancer cells. Findings clearly suggested that the andrographolide-mediated upregulation of DR4 was more pronounced in p53-wild type expressing cancer cells [53]. Mechanistically, it was shown that JNK-driven phosphorylation of p53 on 81st Threonine was noted to be critical for p53 stabilization and transcriptional regulation of target genes. ROS generation triggered activation of JNK which post-translationally modified p53 [53]. Collectively, these results demonstrated that andrographolide induced ROS generation, JNK activation, p53 stabilization, and consequent upregulation of DR4. 

Similar mechanism has been reported in prostate cancer cells. TRAIL and andrographolide combinatorially induced regression of tumors in mice xenografted with prostate cancer cells [54]. Andrographolide effectively reduced NF-ҡB/p65 subunit and stimulated the expression of DR4 and DR5 in bladder cancer cells [55]. 

Although the theoretical dimension of a biological approach to select patients appears to be very attractive, the practical issues of accurate and reliable biomarker selection is challenging. Strategically, selection of patients can be based on sensitivity to TRAIL-based therapeutics and/or to select TRAIL-resistant biomarkers which have already been investigated or that may arise as a treatment response. GALNT14/fucosyltransferase 3/6 assay has previously been studied in phase II clinical trials as a useful and promising diagnostic tool for prediction of sensitivity to TRAIL receptor agonists, drozitumab and dulanermin [56,57]. A detailed investigation of restoration of apoptosis by andrographolide in TRAIL-resistant cancers will be helpful in improving the efficacy of TRAIL-based therapeutics. 

## 8. Regulation of TLR4/MYD88/NF-κB

**Figure 5:**
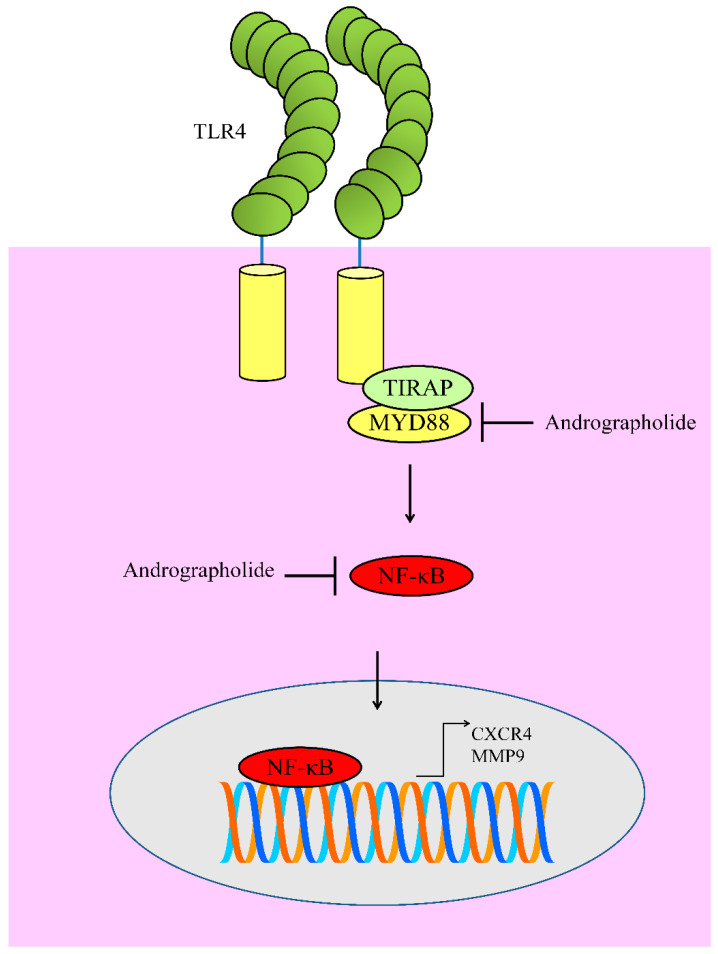
Diagrammatic representation of regulation of TLR4 signaling. Andrographolide inhibited MYD88 mediated activation of NF-κB. Functionally active NF-κB moves into the nucleus to regulate expression of target genes. However, andrographolide inhibited NF-κB activation and prevented its entry into the nucleus.

Toll-like receptors (TLRs) transduce the signals intracellularly by promoting the assembly of signaling adapters MyD88 (myeloid differentiation primary response gene-88). This signalosome was central for the activation of nuclear factor-κB (NF-κB). Andrographolide exerted inhibitory effects on levels of TLR4, MyD8, and p-IκBα in B16 cells. NF-κB has been shown to transcriptionally upregulate CXCR4. Collectively these findings suggested that andrographolide effectively inhibited TLR4/MYD88/ NF-κB/CXCR4 signaling axis in melanoma cells [58] (Figure 5).

Likewise, andrographolide markedly reduced TLR4 and NF-κB to induce apoptosis in myeloma cells [59]. Andrographolide has previously been reported to inhibit NF-κB mediated upregulation of matrix metalloproteinase-9 (MMP9) in colon cancer cells [60]. Molecular studies have provided evidence of the presence of NF-κB binding sites in promoter of MMP9 [61]. 

## 9. Regulation of microRNAs

Since the serendipitous discovery in nematodes, microRNAs (miRNAs) have emerged as highly acclaimed master regulators of cellular and molecular processes. miRNAs have been characterized into tumor suppressor miRNAs and oncogenic miRNAs. After the groundbreaking discovery of miRNAs and their central role in cancer development and progression, scientists have actively pursued the opportunities related to the inhibition of oncogenic miRNAs and overexpression or re-activation of tumor suppressor miRNAs [62,63]. 

The inhibition of oncogenic miRNAs has been studied contextually in different cancers. MDA-MB-231 cells were cultured on chick embryo chorioallantoic membrane to unveil how andrographolide exerted anti-angiogenic effects [64]. Andrographolide attenuated neovascularization in the tumor tissues in a dose-dependent manner. Tissue inhibitor of metalloproteinase (TIMP)-mediated inhibition of metalloproteinases controls a powerful pyramidal cascade inherent to tissue homeostases. TIMP3 is directly targeted by miR-21-5p. Andrographolide repressed miR-21-5p and stimulated the expression of TIMP3 [64]. Therefore, it seems clear that andrographolide interferes with angiogenesis mainly through the inhibition of miR-21-5p and upregulation of TIMP3. 

Andrographolide regulated miRNAs through transcriptional factors p53 and HNF4A (Hepatocyte nuclear factor-4-alpha) [65]. Andrographolide induced an increase in p53 levels that further stimulated expression of its target miRNAs (miR-181a, miR-17and miR-224). p53 upregulation markedly reduced glutathione peroxidase (miR-181a) and thioredoxin interacting protein (miR-17, miR-224) [65]. Andrographolide exerted inhibitory effects on HNF4A levels that further downregulated miR-377 and miR-433. HNF4A downregulation resulted in an increase in glutathione cysteine ligase (miR-433] and heme oxygenase-1 (miR-377) [65]. These findings further supported the notion that andrographolide controlled tumor suppressor and oncogenic miRNAs through transcriptional factors. 

Andrographolide was found to be effective against ALDH1^+^CD44^+^ oral cancer stem cells (OCSCs) isolated from patient-derived cell lines [66]. Andrographolide concentration-dependently decreased ALDH1 activity and also reduced the percentage of CD44^+^ cells. Andrographolide induced regression of tumors in immuno-deficient nude mice injected subcutaneously with OCSCs. Bmi1 played critical role in self-renewal capacity of OCSCs [66]. miR-218 directly targeted Bmi1 and significantly reduced self-renewal capacity of OCSCs. Andrographolide stimulated the expression of miR-218 in OCSCs [66]. Overall, these aspects highlighted the potential of andrographolide to suppress OCSCs through activation of miR-218. 

It is interesting to know that andrographolide inhibit EMT in mouse lung tissues. Cigarette smoke induced EMT in mouse lung tissues and human bronchial epithelial cells [67]. Detailed mechanisms were clearly revealed that cigarette smoke induced the upregulation of a long non-coding RNA HOTAIR and EZH2 (enhancer of zeste homolog-2). Andrographolide exerted repressive effects on HOTAIR and EZH2 in lung tissue of cigarette smoke-exposed mice [67].

## 10. Concluding Remarks

Andrographolide is gaining attention because of its remarkable potential to modulate different signaling cascades in different cancers. However, these findings have just started to open new horizons for a detailed and critical preclinical investigation of the abilities of andrographolide to therapeutically target deregulated signaling pathways and simultaneously inhibit tumor growth in xenografted mice. With the continuous refinement of existing scientific evidence related to the chemopreventive role of andrographolide in different cancers, more complex and intriguing questions have started to challenge molecular and cancer biologists. Andrographolide has been shown to modulate STAT3 but it will be essential to evaluate full spectrum of STAT proteins alongwith positive and negative regulators of STAT signaling to fully validate role of andrographolide as an ideal STAT inhibitor. Likewise, there are scattered studies about the regulation of Wnt/β-Catenin signaling by andrographolide in different cancers. The therapeutic targeting of VEGF/VEGFR mediated signaling has remained complicated and andrographolide and its derivatives have sparked the interest of researchers for the further analysis of andrographolide efficacy against different growth factor-mediated cascades. Andrographolide has been tested to restore TRAIL-induced apoptosis in different cancers. However, these findings need further research to unlock how andrographolide restored apoptosis in TRAIL-resistant cancers. In addition, the regulation of non-coding RNAs by andrographolide in different cancers is incompletely investigated. The use of systems biology may be helpful to realistically analyze the regulation of non-coding RNAs, the interaction of miRNA and mRNAs, and how different non-coding RNAs transcriptionally regulated different genes. 

## 11. Future Prospects

Andrographolide mediated targeting of Notch, SHH/GLI, and TGF/SMAD have not been comprehensively explored. Successful development of Notch pathway-targeting agents will require a mechanistic knowledge of the functions of Notch signaling in specified cancers. Likewise, the regulation of TGF/SMAD signaling needs to be extensively explored because of tight connection between hyperactive TGF/SMAD signaling and EMT. Andrographolide repressed RANKL-induced NF-ҝB signaling and consequently inhibited cancer-induced bone loss in tumor bearing mice [68]. Emerging technologies, such as synthetic biology and genomics, have opened new horizons for the identification of medicinal properties of biologically active chemicals from natural sources. The identification of natural products which possess remarkable pharmacological properties may provide an alternative option for anti-cancer treatment.

## Abbreviations

4EBP	eIF4E-binding protein
AMPK	AMP-activated Protein Kinase
ASC	Apoptosis-associated Speck-like Protein containing a CARD
BMDM	bone marrow-derived macrophages
CARD	Caspase Recruitment Domain
DR4 and DR5	death receptors
EMT	epithelial to mesenchymal transition
ERK1/2	extracellular signal regulated kinase-1/2
EZH2	enhancer of zeste homolog-2
GAP	GTPase-activating protein
HCC	hepatocellular carcinoma
HNF4A	Hepatocyte nuclear factor-4-alpha
IL-6	interleukin-6
JAK/STAT	Janus Kinase/Signal Transducer and Activator of Transcription
LKB1	liver kinase B1
p-LKB1/STK11	Liver kinase B1 or Serine/threonine kinase-11
MyD88	myeloid differentiation primary response gene-88
MMP9	matrix metalloproteinase-9
miRNAs	microRNAs
mTOR	mammalian Target of Rapamycin
NF-κB	nuclear factor kappa B
MAPKs	mitogen-activated protein kinases
OCSCs	oral cancer stem cells
PDK1	Phosphoinositide-dependent kinase 1
PIAS	Protein Inhibitors of Activated STATs
PYD	PYRIN domain
RAPTOR	regulatory-associated protein of mTOR
ROS	reactive oxygen species
S6K	ribosomal protein S6 kinase
SOCS	Suppressor of cytokine signaling
TCF/LEF	T-cell factor and lymphocyte enhancing factor
TGF	transforming growth factor
TIMP	Tissue inhibitor of metalloproteinase
TLRs	Toll-like receptors
TRAIL	Tumor necrosis factor-related apoptosis-inducing ligand
VEGFs	vascular endothelial growth factors
VEGFRs	vascular endothelial growth factors receptors
